# Observation on the efficacy of sublingual immunotherapy with dust mite allergen for perennial allergic rhinitis and the mechanism of action on ILCs with ILC1s and ILC2s and ILC3s

**DOI:** 10.1097/MD.0000000000032019

**Published:** 2022-12-02

**Authors:** Guangjun Tang, Youxing Lan, Bo Do, Jiafeng Lu, Kai Yang, Long Chai, Fangming Chen, Li Tian

**Affiliations:** a Department of Otolaryngology, Hospital of Chengdu University of Traditional Chinese Medicine, Chengdu, China; b Department of Otolaryngology Head and Neck Surgery, Peoples Hospital of Anshun City Guizhou Province, Guizhou, China.

**Keywords:** curative effect, discussion on mechanism, perennial allergic rhinitis, sublingual immunotherapy

## Abstract

Allergic rhinitis (AR) is considered to be 1 of the most difficult diseases to treat globally. It has a serious impact on the quality of life and social economy of patients and has become an important global health problem. Several drugs have been recommended to treat AR, but their effectiveness and mechanism of action in these patients remain unclear. The purpose of this study will be to compare the efficacy and mechanism of action of 2 drugs for the treatment of AR (moderate to severe): a Dermatophagoides Farinae Drops Sublingual Immunotherapy and a Momethasone Furoate nasal spray as an adjunct to the treatment of subjects with AR.

**Methods:**

A randomized, prospective, double-blind (patient and evaluator) clinical trial. The participants (n = 60) will be randomly distributed into 2 groups. The experimental group will receive a sublingual Immunotherapy for 3 months. The control group will receive the mometasone furoate nasal spray for 3 months. Before treatment, 1 month and 3 months after treatment, total nasal symptom score scale, Visual analogue Scale and Quality of Life questionnaire of rhinoconjunctivitis will be measured and Changes of the serums of IgE, interferon-γ, IL-4, IL-17, tumor necrosis factor-α, IL-5, IL-9, IL-13, IL-25, IL-33, vascular endothelial growth factor, TSLP and IL-22 in both groups. The measurements will be performed by the same researcher who was unaware of the participants’ subgroup.

**Discussion:**

We believe that the treatment of perennial AR with sublingual Immunotherapy and nasal hormones will be more effective in these patients. Furthermore, the sublingual Immunotherapy mainly acts mostly on the cellular immunity, while nasal hormones mainly act on local inflammatory responses. We expect to clarify which treatments are more effective and how they work in improving perennial AR.

## 1. Introduction

Allergic rhinitis (AR) is a common and frequent ailment of otolaryngology. It is a perennial or seasonal disease with symptoms such as sneezing, runny nose, itching nose, nasal congestion and anosmia.^[1]^ Numerous epidemiological studies have been shown that AR affects 10% to 40% of the global population.^[2]^ It has become a major chronic inflammatory respiratory disease and is considered 1 of the most difficult diseases to treat globally.^[3]^

Although AR is not a serious disease, but due to its recurrent, persistent, and complicating characteristics,^[4–6]^ it has a serious impact on the quality of life and socio-economic well-being of patients and has become a major global health problem.^[7]^ It is reported that economic losses in EU countries can be between 30 and 50 billion euros per year.^[8]^ A large sample study in the United States showed that AR reduced worker productivity more than any other disease, including hypertension, diabetes, and heart disease.^[9]^

The treatment of AR mainly includes environmental control, drug therapy, immunotherapy, etc.^[9,10]^ all kinds of treatment methods have their advantages. However, there are no good measures to avoid dust mite allergy at present. Therefore, it is becoming more and more important for scholars to change patients’ allergic physique through specific immunotherapies.

AR is a noninfectious chronic inflammatory disease of the nasal mucosa mainly mediated by immunoglobulin E after exposure to allergens in atopic individuals.^[11]^ Regulating the immune balance of Th1/Th2/Th17 cells during the occurrence and development of AR is considered to be an important approach in the current treatment of AR. But there is increasing evidence that innate immune response is also the pathogenesis of AR. Innate lymphoid cells (ILCs) are involved in mucosal immune formation, lymphocyte development, tissue damage repair and epithelial barrier protection, and play an important role in fighting infection, regulating inflammation and maintaining immune homeostasis. Some studies have proposed that the 3 subsets of innate lymphocytes (ILC1s, ILC2s, ILC3s) are functionally approximately corresponding to Th1, Th2, and Th17 of T helper cells. However, current studies have not been conclusive, and the specific mechanism of ILCs in the development of AR has not been fully elucidated.

Our objective was to compare the efficacy of sublingual immunotherapy and nasal hormone in the treatment of perennial AR and the mechanism of action on ILCs, and to reveal the correlation between ILCs (ILC1s, ILC2s, ILC3s) and Th1/Th2/Th17 cell immunity, so as to provide research basis for clinical study of AR.

## 2. Methods

### 2.1. Study design

**Figure 1.**
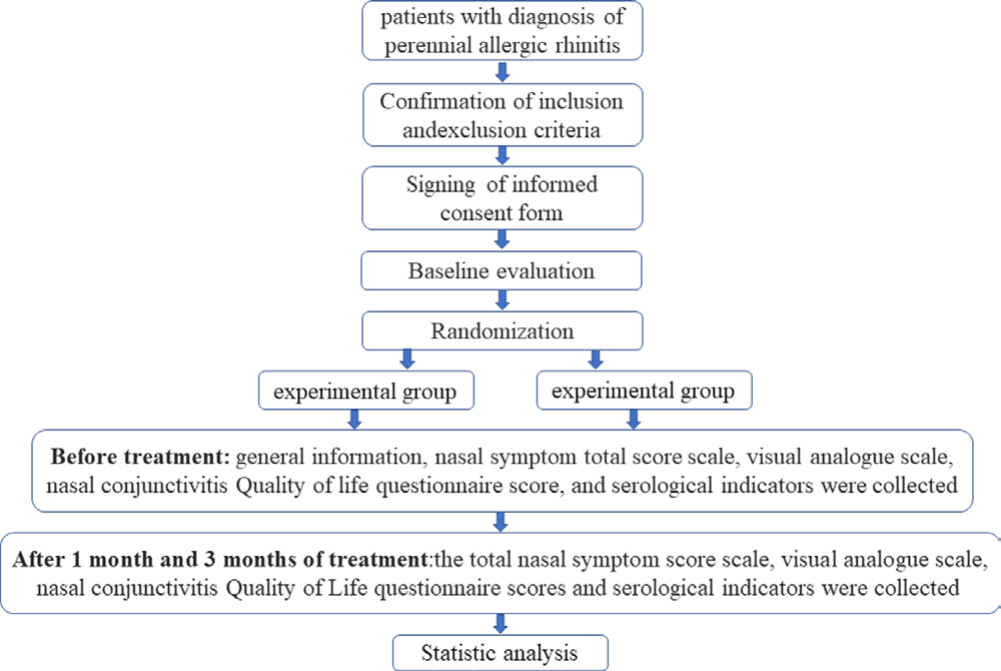
Is the technology roadmap.

A randomized, prospective, double-blind (patient and evaluator) clinical trial. The Researchers should not be blinded due to the use of therapeutic method. Figure 1 shows a diagram with the different phases of the study.

### 2.2. Study population. Sample recruitment

We will recruit and include 60 participants with perennial AR from the outpatient Department of Otorhinolaryngology Head and Neck Surgery Sant by the People’s Hospital of Anshun City of Guizhou Province (China). All participants receiving this service must be seen by a physician (allergy subspecialist or general physician). All those diagnosed with perennial AR (of more than 1 years duration) according to the Guidelines for AR^[1,12,13]^ during that appointment will be referred to a Outpatient physician of otolaryngology Head and neck Surgery, who will check whether they meet the other inclusion and exclusion criteria before offering them the opportunity to participate in the study.

The inclusion criteria will be: We will include participants with perennial AR only (not seasonal AR); above 18 years old to below 65 years old; both sexes; The participants has not received specific immunotherapy in the past 1 month or has not used any drugs to treatment the AR in the past 1 week; The participants must be examined with a positive result in the IgE sensitization test to aeroallergens (skin prick test or serum allergen specific IgE); they must with normal cognitive function and agree to participate in this study and sign the informed consent form.

The exclusion criteria will be: Participants who met any of the following criteria were excluded: The participants with severe nasal septum deviation, chronic rhinosinusitis, bronchial asthma, nasal polyps, upper respiratory tract infection, lung infection and other diseases; with severe dysfunction of heart, liver, kidney or autoimmune diseases; Pregnant or lactating women; Allergic constitution and allergic to the experimental drugs and ingredients; With drug addiction history; With major neuropsychiatric diseases who cannot take medication regularly; participating in other clinical trials.

The participants who meet all inclusion criteria and not meet exclusion criteria will have the opportunity to participate in the study and provide all relevant information orally and in writing. They will be told to be randomly assigned to study groups, each of which will be treated with a different drug, all of which are appropriate for their condition, and the goal of the study is to determine which drug produces the best results.

To maintain this blindness, the participants will not be told which group they had been assigned to.

If they decide to participate, they will be asked to sign an informed consent form, and the recruiting physician will conduct a baseline assessment.

### 2.3. Randomization

We will be collected 60 subjects and randomize them into 2 groups (the experimental group and control group.) and use a computerized randomization method using EXCEL software and RAND function to generate random numbers (a total of 60 random numbers). Sixty randomly selected subjects will have a 1/2 chance of being randomly assigned to either the experimental or control groups. According to the order of presentation, participants will be assigned to the corresponding group for treatment according to the generated random number table.

The ENT clinic practitioner performing the intervention will be the only 1 with access to the list generated by the computer program. This physician cannot be blinded by using manual techniques, and he/she will know which group the subject is assigned to by referring to the number assigned by the evaluator.

To ensure the concealment and blindness of the task, the subjects will not know which group they have been assigned to.

### 2.4. Variables studied

The recruiting physician (PhD in otolaryngology with 8 years of experience in the field) will perform all assessments for the study and remain blinded to the group to which subjects are assigned at all times.

Table 1 shows the sociodemographic variables and the values that each 1 may have. The following variables: sex, age, occupation, contact information, duration of symptoms, allergic history and Genetic history will be recorded at the beginning of treatment by anamnesis.

**Table 1:** Independent sociodemographic variables.

Variable	Values
Sex	Man/Woman
Age	Years
Occupation	What kind of job
Contact information	Phone number
Duration of symptoms	Daily – Weekly – Monthly – Quarterly – Annual
Allergic history	Yes/No
Genetic history	Yes/No

Table 2 shows the outcome variables, the evaluation period for each variable, and the measurement tools used. All assessments will be performed by a physician, who will also be responsible for recruitment, and the group assigned to each subject will be assessed in a blinded manner throughout the assessment period.

**Table 2:** Outcome variables, the evaluation period for each variable.

Outcome variables	Evaluation period			Measuring instrument
	Baseline	1 mo post-treatment		3 mo post-treatment
Nasal symptoms and signs score	X	X	X	TNSS
Intensity of nasal pain (main symptom)	X	X	X	VAS
Rhinitis Quality of Life Questionnaire	X	X	X	RQLQ
Serological test	X	X	X	The level of serological indicators

RQLQ = rhinoconjunctivitis quality of life questionnaire, TNSS = the total nasal symptom score, VAS = Visual analog scale.

The primary outcome indicator is the decrease in the total nasal symptom score^[14]^ (including self-assessment of nasal congestion, runny nose, itchy nose, and sneezing) and Changes in the level of serological indicators (Enzyme-linked immunosorbent assay will be used to detect). The secondary outcome indicators are visual analogue scale and Standardized Rhinoconjunctivitis Quality of Life Questionnaire^[15]^ and mitigate changes in drug use. Data will be recorded by Case report forms (CRFs) before treatment, at 1 month of treatment, and at 3 months of treatment.

### 2.5. Adverse events and termination of trial

For safety reasons, all unexpected reactions related to the study treatment will be recorded and described in detail, including time to onset, duration of symptoms, severity of symptoms, management measures, time to resolution of adverse effects, and causal classification. All adverse events, whether or not they are related to the study treatment, will be recorded and managed by the investigator. If more than 25% of the patients discontinued the intervention because of adverse events, the superior physician would decide to discontinue the trial. The CRF will be used for data collection to document demographics, assessment, and reasons for patient withdrawal. At the end of the study, the investigator will submit a CRF form to the data Management Committee.

### 2.6. Data collection and management

Data for this study will be collected by 2 assistants and entered into pre-designed forms. Information and data related to this study will be collected, shared, and stored in a separate repository to protect confidentiality before, during, and after testing. The information was not available to anyone outside the research team. Participants’ study information may not be released outside the study without their written permission.

### 2.7. Statistical analysis data

SPSS (Statistical Product Service Solutions) is the earliest statistical analysis software in the world. Norman H. Nie, C. Hadlai (Tex) Hull and Dale H. Bent, three graduate students from Stanford University in the United States, successfully researched and developed the software in 1968. Meanwhile, SPSS company was established. In 1975, SPSS headquarters was established as a corporate organization in Chicago.

On July 28, 2009, IBM announced it would acquire SPSS, Inc., a provider of statistical analysis software, for $1.2 billion in cash. The latest version of SPSS is now 25 and renamed IBM SPSS Statistics. So far, SPSS company has more than 40 years of growth history.

### 2.8. Study monitoring

The hospital’s Clinical Trials Administration will be responsible for overseeing the progress of the study, and this organization will have no involvement in the study at all and will not present a conflict of interest. On-site monitoring meetings will be hold once before and 1 month after the start of the study to monitor the progress of the study and to ensure compliance with established protocols, good clinical practice guidelines and applicable regulatory requirements. Although a separate data monitoring committee will not be established, the hospital clinical trial administration will perform this function by regularly monitoring the clinical trial to ensure scientific validity, scientific integrity, and data accuracy.

## 3. Discussion

AR is a noninfectious chronic inflammatory disease of nasal mucosa mainly mediated by immunoglobulin E after atopic individuals are exposed to allergens. AR can be classified as seasonal or perennial according to the type of allergen. According to the course of disease can be divided into intermittent and persistent; The impact on quality of life is divided into mild and moderate-severe.^[1]^ Current studies have shown that Th1/Th2/Th17 cell immune imbalance is an important mechanism of AR pathogenesis.^[16–18]^

In the development of AR, T cells are the only cells that react directly with antigens. Helper T cells are derived from interleukin-2-producing precursor cells. After initial stimulation, these cells develop into Th0 cells (CD4 + T cells), which produce cytokines including IFN-γ, IL-2, IL-4, and IL-5. Th0 cells differentiate into Th1 cells under the induction of IL-12 and IFN-γ, which secrete IFN-γ, IL-2 and tumor necrosis factor-β and participate in cellular immune response. Under the induction of IL-4, they differentiate into Th2 cells and secrete IL-4, IL-5, IL-13, IL-8 and other cytokines to participate in humoral immune response.^[19]^ Th17 is a new type of T helper lymphocyte, which is a pro-inflammatory cell that can activate the body’s inflammatory response and participate in the regulation of the autoimmune system. It was discovered in 2003 and got its name because it can secrete iconic factors such as IL-17 and IL-23, and plays an important role in the body’s self-immune response.^[20]^

In the occurrence and development of AR, IFN-γ, IL-4 and IL-17 are the main effectors of Th1, Th2 and Th17 respectively.^[21]^ It has been reported^[22]^ that IL-4 immune inflammatory factor released by Th2 cells has a regulatory effect on the level of IgE. However, IFN-γ released by Th1 cells has an inhibitory effect on IL-4 secretion by Th2 cells.^[23]^ IL-17 is a cytokine secreted by Th17 cells with strong proinflammatory effect. Serum IL-17 in patients is positively correlated with IgE level, and its increased level can be used as an indicator for the diagnosis of AR.^[24]^ Therefore, regulating the immune balance of Th1/Th2/Th17 cells is an important way to treat AR. However, increasing evidence shows that innate immune response is also the pathogenesis of AR.

The innate immune system is the first line of defense against invading pathogens or antigens, and its response is rapid and nonspecific. Subsequently, the activated adaptive immune system performs complete elimination of specific antigens.^[25]^ ILCs, as an important effector cell population of innate immunity, are characterized by 3 major characteristics: they do not undergo receptor gene rearrangement and clonal selection, lack of phenotypic markers of myeloid cells and dendritic cells, and their morphology belongs to the lymphoid lineage. ILCs are mostly tissue-resident lymphocytes, mainly distributed in the tonsil, broncho-lung, intestinal tract, skin and other mucosal barrier sites. Ilcs are involved in mucosal immune formation, lymphocyte development, tissue damage repair and epithelial barrier protection, and play an important role in fighting infection, regulating inflammation and maintaining immune homeostasis.^[26]^

According to the phenotype and cytokines secreted by ILCs, ILCs can be divided into 3 subsets of type 1, 2 and 3 innate lymphocytes (ILC1s, ILC2s and ILC3s),^[27]^ which are functionally approximately corresponding to Th1, Th2 and Th17 of Helper T cells. ILC1s includes natural killer cells (NK) and ILC1 cells, which depend on T-box transcription factor and produce large amounts of IFN-γ and tumor necrosis factor-α. The development of ILC2s depends on the transcription factor GATA3 to produce Th2-type cytokines and other effector molecules, such as IL-4, IL-5, IL-9, IL-13 and Vascular endothelial growth factor, which drive the development of type 2 immune response. Moreover, unlike T cells, which recognize specific antigens, ILC2s respond to nonspecific cytokines, including IL-25, IL-33, and Thymic stromal lymphocytes produce hormone. TSLP can stimulate the activation and proliferation of ILC2s to produce a large amount of IL-5 and IL-13, resulting in airway inflammation and airway hyperresponsiveness. ILC3s depend on the transcription factor RORTt to produce cytokines IL-17 and IL-22 similar to Th17.^[27–29]^

Some studies have found that after the nasal epithelium of AR patients is stimulated by allergens, the pro-inflammatory cytokines in the epithelium increase, and IL-25, IL-33 and TSLP can be detected in the nasal lavage fluid of patients with house dust mite allergy.^[30,31]^ However, the level of IL-25 released by peripheral blood mononuclear cells will be up-regulated after basophils of birch and pollen allergy patients are stimulated by allergens.^[32]^ Other studies have shown that the number of ILC2 in peripheral blood of patients with house dust mite allergy is increased, and its number change is positively correlated with the severity of symptoms.^[33,34]^ Studies on AR caused by plant allergens found that during the grass pollen season, the number of ILC2 and ILC3 in peripheral blood of patients with grass pollen allergy increased, while the number of ILC1 did not change significantly.^[35]^ However, Bartemes et al^[36]^found that the number of ILC2 in peripheral blood of AR patients did not increase, but that of asthma patients increased. There is no consensus on whether the number of ILC2s in peripheral blood of AR patients is increased.

In conclusion, the mechanism of AR is mainly related to the imbalance of Th1/Th2/Th17 cell immunity, but more and more evidence shows that innate immune response is also the pathogenesis of AR, and the specific mechanism of ILCs in the development of AR has not been fully elucidated.

Therefore, this study aims to explore the efficacy of sublingual desensitization in the treatment of perennial AR and its mechanism of action on ILCs, reveal the correlation between ILCs (ILC1s, ILC2s, ILC3s) and Th1/Th2/Th17 cell immunity, and provide research basis for clinical research on AR.

## Author contributions

**Conceptualization:** Guangjun Tang, Bo Do.

**Data curation:** Kai Yang, Youxing Lan.

**Formal analysis:** Jiafeng Lu.

**Funding acquisition:** Guangjun Tang.

**Software:** Long Chai.

**Supervision:** Fangming Chen.

**Writing – original draft:** Guangjun Tang, Youxing Lan.

**Writing – review & editing:** Guangjun Tang, Li Tian.
